# Top-Down System for Multi-Person 3D Absolute Pose Estimation from Monocular Videos

**DOI:** 10.3390/s22114109

**Published:** 2022-05-28

**Authors:** Amal El Kaid, Denis Brazey, Vincent Barra, Karim Baïna

**Affiliations:** 1 Université Clermont-Auvergne, CNRS, Mines de Saint-Étienne, Clermont-Auvergne-INP, LIMOS, 63000 Clermont-Ferrand, France; vincent.barra@limos.fr; 2 Alqualsadi Research Team, Rabat IT Center, ENSIAS, Mohammed V University in Rabat, Rabat 10112, Morocco; karim.baina@ensias.um5.ac.ma; 3 Société Prynel, RD974, 21190 Corpeau, France; dbrazey@pryntec.com

**Keywords:** 3D multi-person pose estimation, absolute poses, camera-centric coordinates, computer vision, artificial intelligence, deep-learning

## Abstract

Two-dimensional (2D) multi-person pose estimation and three-dimensional (3D) root-relative pose estimation from a monocular RGB camera have made significant progress recently. Yet, real-world applications require depth estimations and the ability to determine the distances between people in a scene. Therefore, it is necessary to recover the 3D absolute poses of several people. However, this is still a challenge when using cameras from single points of view. Furthermore, the previously proposed systems typically required a significant amount of resources and memory. To overcome these restrictions, we herein propose a real-time framework for multi-person 3D absolute pose estimation from a monocular camera, which integrates a human detector, a 2D pose estimator, a 3D root-relative pose reconstructor, and a root depth estimator in a top-down manner. The proposed system, called Root-GAST-Net, is based on modified versions of GAST-Net and RootNet networks. The efficiency of the proposed Root-GAST-Net system is demonstrated through quantitative and qualitative evaluations on two benchmark datasets, Human3.6M and MuPoTS-3D. On all evaluated metrics, our experimental results on the MuPoTS-3D dataset outperform the current state-of-the-art by a significant margin, and can run in real-time at 15 fps on the Nvidia GeForce GTX 1080.

## 1. Introduction

**Figure 1:**
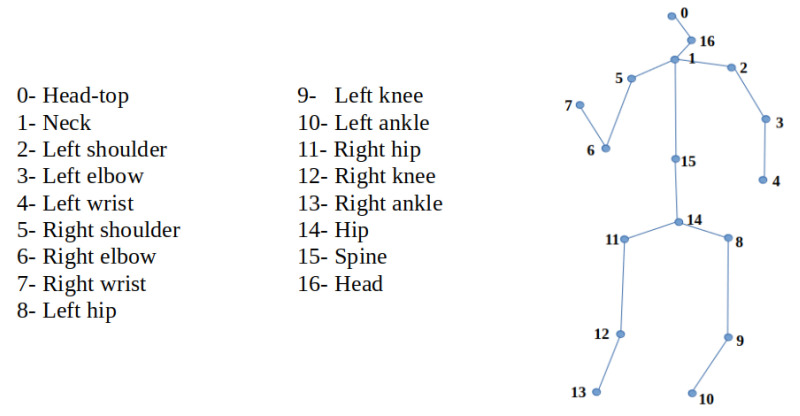
3D Skeleton model in MuPoTS-3D format and joints names.

Human pose estimation (HPE) is a popular task in computer vision. It aims to predict and track the location of joints (e.g., elbow, wrist) or body parts of one or more human bodies; it associates them with segments in graphical form (from an image or sequence of images) to represent the human’s orientation and it describe the actual posture. This is an important process for understanding human behavior and human–computer interactions. An example of a human posture skeleton is illustrated in Figure 1.

With human pose estimation, tracking a person or multiple people in real space can be done at an incredibly granular level. This powerful capability unlocks a wide range of industrial applications [1,2,3,4,5,6,7,8], including gaming, animation, motion transfer, augmented reality, human–robot cooperation and training, biomechanical analysis for medical/healthcare, sports fields, gesture control, autonomous driving, human fall detection, action prediction, security and surveillance, etc.

Pose estimation can be performed in two ways: in a two-dimensional space to predict XY image coordinates or in a three-dimensional space to predict the XYZ camera or world coordinates. However, most real-life applications require depth estimation, which provides informative knowledge since 2D poses are often confusing. They can appear identical when in fact they represent completely distinct poses. This makes activity recognition difficult and leads researchers to employ 3D pose estimation.

Recently, 3D root-relative human pose estimation has shown remarkable progress. Several methods [9,10,11,12,13,14] propose alleviating the problem by using multi-view images or videos as input. However, multi-view observations are expensive to obtain in daily life scenarios. Thus, the use of 3D human pose estimations from monocular images or videos is in high demand. State-of-the-art approaches that use monocular data [15,16,17,18,19,20,21,22] usually decouple the problem into two main phases: 2D pose estimation for joint detection and localization in the image space, and then lifting of the 2D pixel coordinates to 3D keypoint-position predictions in the camera space. In our research, we followed the same strategy and focused on the second phase, i.e., the 3D pose reconstruction from a sequence of 2D keypoints. Two-dimensional (2D) pose estimation is a popular vision problem that has been studied in many works, e.g., [23,24,25,26,27,28] and has been greatly improved especially using the deep learning paradigm.

Indeed, 3D pose estimation approaches show promising results on single-person datasets, such as Human3.6M [29] and HumanEva-I [30]. However, they do not perform well in multi-person scenarios, which are the most common cases in real-world applications and surveillance systems. The distances between people can be crucial in the analysis and recognition of their interactions. This introduces the absolute pose [31,32,33], which aims to locate the root joint (key central point of the person) and estimate its distance from the camera. At present, the 3D multi-person pose estimation still faces a great challenge. When possible, stereo vision calibration is used to determine the exact position of a person from images taken from different points of view. However, these kinds of data are not always available, and they significantly raise the overall costs of the process procedures. Moreover, acquiring such data is impractical in real-time system applications, as we seek to optimize the amount of data that must be captured and processed. This shows the gap between scientific literature and real-world requirements.

The purpose of this study was to present a framework that could accomplish more accurate and robust 3D multi-person pose estimations from a monocular video, from these circumstances and industrial constraints. Thus, we propose an integrated top-down approach that combines GAST-Net for reconstructing 3D root-relative keypoints from 2D keypoints and RootNet for estimating root depth from human bounding boxes. It generates an appropriate 3D multi-skeleton estimation result from a monocular video while maintaining low computational costs and short execution times.

Basically, the system is the result of a series of improvements that boost accuracy by more than 8.8 percentage points on 3D-PCK_*abs*_ on the MuPoTS-3D [34] dataset, when compared to the approaches in the literature [31,32,33,35,36].

**Figure 2:**
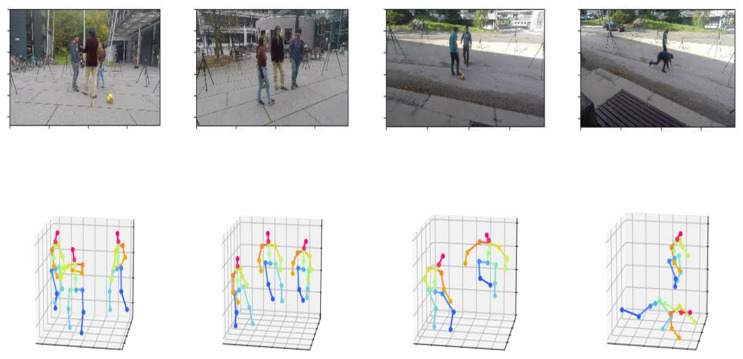
Examples of 3D absolute poses resulting from our whole framework.

Examples of results from our whole framework are illustrated in Figure 2.

The main contributions of this work can be summarized as follows:

- The proposal of an integrated top-down framework based on a modified GAST-Net and RootNet networks for multi-person 3D pose estimation from a monocular RGB video in a short execution time.
- Outperforming existing 3D multi-person absolute pose estimation methods in a MuPoTS-3D dataset by more than 8.8 percentage points on 3D-PCK*_abs_* and by more than 12.6 percentage points on AP${}_{25}^{root}$.

The paper is organized as follows. Section 2 illustrates the review of conventional literature on 3D pose estimation based on different levels: the input type (video), the number of instances (multi-person), and the approach following the 3D root-relative pose estimation (two-stage approach). Section 3 demonstrates the proposed framework methodology. Section 4 explains the implementation details, the results and discussion. Section 5 provides a conclusion of the work.

## 2. Related Works

### 2.1. Two-Stage Pose Estimation

Several works [22,37,38,39,40,41,42,43] apply deep neural networks on 3D pose estimation tasks to learn the direct mapping between RGB images and their corresponding 3D poses in one stage. However, this needs labeled data for supervised training, usually impractical out of MoCap labs. Unsupervised learning algorithms require sophisticated architectures with high computation costs, which are impractical too in realistic applications. To this end, Martinez et al. [44] introduced a two-stage prediction approach. They first predicted the 2D pose from the image and then lifted 2D joint coordinates to the 3D space via a fully connected residual network. Fang et al. [45] introduced a model to encode the mapping function of the human pose from 2D to 3D by explicitly encoding the human body configuration with pose grammar. To improve the generalization of the trained 2D-to-3D pose estimator, Gong et al. [46] proposed a pose augmentation framework (PoseAug) exploiting a differentiable augmentation module based on a neural network. In Ref. [47], the authors created a shape dictionary by collecting all 3D poses in the training set to be aligned by the Procrustes method, to concisely summarize the variability in training data and enable a sparse representation. A convex approach was then proposed to jointly estimate the coefficients of the sparse representation. The same authors [48] predicted the uncertainty heatmaps of the 2D joint locations, then combined these maps with a sparse model of a 3D human pose to retrieve the 3D pose via an EM algorithm. Ref. [49] adopted a large library of 2D keypoints and their 3D representations to match the depths of the 2D poses estimated by the k-nearest neighbor algorithm. Hossain et al. [50] proposed two 2-layered normalized LSTM networks with residual connections to leverage temporal information for lifting 2D joint locations to 3D positions.

### 2.2. Video Pose Estimation

Although 3D coordinates can be determined from a single image, temporal algorithms used in videos have better accuracies than simple frame-by-frame approaches. Most works deploy recurrent neural networks (RNNs) [50,51] to exploit temporal information. Long short-term memory networks (LSTMs) [52] are the most widely used RNN architectures for learning long-term dependencies in pose estimation problems because of their ability to preserve information over time. In [51], propagating LSTM networks (p-LSTMs) were proposed to estimate depth information from 2D keypoints. Ref. [53] presented a two-part spatial–temporal convolutional LSTM model (ST-CLSTM) to capture spatial features and temporal consistency between frames. The authors used ST-CLSTM as the generator and a 3D CNN as the discriminator to output the temporal loss from the estimated and ground truth depth sequences. AnimePose [54] used Scene-LSTM to estimate the person’s temporal trajectory and track overlapping postures in obscure frames based on their predictions in prior frames. Temporal convolutional networks (TCNs) [55], on the other hand, give additional benefits, such as convolution sharing and low memory requirements for training; this is very advantageous when dealing with extended input sequences. TCN evaluation and training are hence faster than with RNN. As a result, they are becoming increasingly employed in pose estimation [35,37,38,39,56], especially in real-time systems [57,58]. Moreover, Ref. [39] proposed employing dilated temporal convolutions in a fully convolutional model; moreover, [59] used it as an automatic framework for semantic motion segmentation. Li et al. [60] captured long-range dependencies using transformer-based architecture.

### 2.3. Spatial–Temporal Graph Convolution Network

Despite the acquired temporal information’s ability to anticipate smoother poses, the depths and self-occlusions remain ambiguous. A graph convolutional network (GCN) was used to exploit the spatiotemporal information that allowed to lower these ambiguities. GCNs have greatly improved 3D human pose estimations by representing the human skeleton as an undirected graph. The spatial–temporal graph convolutional network (ST-GCN) [61] was the first approach to use graph CNNs for skeleton-based action recognition. Zhou et al. [22] developed the semantic graph convolutional network (SemGCN) for the 3D human pose regression challenge. The SemGCN aims to learn by capturing semantic information, such as local and global node relationships through end-to-end training. The graph attention spatiotemporal convolutional network (GAST-Net) [57] also combines common convolutional networks to integrate the spatiotemporal information. GAST-Net comprises two types of graph attention blocks: a local spatial attention network (to model the hierarchical and symmetrical structures of the human skeleton) and a global spatial attention network (to extract global semantic information and better encode the human body’s spatial characteristics). Cai et al. [62] developed an undirected graph to model the spatial–temporal connections between distinct joints for 3D single-person pose estimation from video data. In Ref. [32], the authors utilized a graphical neural network (GNN) to efficiently aggregate the features corresponding to the different types of articulation, where each type was represented by a graph node. The GCNs based on directed graphs were also adopted by Cheng et al. [35] to model human joint GCNs that refine potentially imperfect poses obtained from 2D pose heatmaps, and human bone GCNs, to model bone connections. The authors also used two TCNs to estimate the 3D root-relative pose and the absolute root depth. Finally, the dynamic graph convolutional module (DGCM) [63] applied GCN for a multi-person 2D pose estimation framework.

### 2.4. Multi-Person 3D Pose Estimation

Only a few studies were conducted on 3D multi-person pose estimation from a single RGB image. Generally, existing methods can be divided into two categories: top-down and bottom-up approaches.

Top-down 3D human pose estimation methods [64,65,66] commonly use human detection as an essential part to crop each person in a bounding box and then estimate person-centric 3D full-body joints [31,39,58]. These methods show promising performances, but their main drawbacks still involve the independent detection and process of each person. Hence, they are likely to suffer from inter-person occlusions and close interactions. Rogez et al. [65,67] introduced LCR-Net, which classified bounding boxes generated into a set of K-poses, refined using a regressor. The architecture contains three stages that share the convolutional feature layers and are jointly trained. Likewise, Benzine et al. [68] proposed the pose estimation and detection anchor-based network (PandaNet), an anchor-based single-shot approach. The network predicts the 2D/3D pose regression into a single forward pass for each bounding box detected in a given image.

To predict camera-centric, Moon et al. [31] processed each cropped person’s image independently. They produced root-relative 3D joints using PoseNet [21] and estimated the pelvis keypoint localization of each person using the RootNet model. Similarly, hierarchical multi-person ordinal relations (HMOR) [69] is a coarse-to-fine architecture that hierarchically estimates multi-person ordinal relations through instance-level, part-level, and joint-level. The end-to-end HDNet architecture [32] follows the same pipeline, extract pose, and depth data using a pyramidal feature network [70] as the backbone. Features are then propagated and aggregated using GNN for target depth estimation. In [35], after obtaining the 2D poses from the 2D pose estimator, the poses were normalized to be centered on the root point. Then, the authors used three temporal models—joint-TCN, root-TCN, and velocity-TCN—to obtain absolute 3D human poses, but on monocular videos instead of single images.

On the other hand, bottom-up approaches [34,71,72] first produced all body joint locations and depth maps, then associated body parts to each person according to the root depth and part relative depth. Mehta et al. [34] proposed a single forward pass regardless of the number of people in the scene. The authors applied temporal and kinematic constraints in three steps to predict occlusion-robust PoseMaps (ORPM) and part affinity fields [27]. Another bottom-up multi-stage framework was proposed by Zanfir et al. [73], which first estimated the volumetric heatmaps to determine the 3D keypoint locations and limbs using the confidence scores of all possible connections, and then conducted skeleton grouping in order to assign limbs to various people. Likewise, Fabbri et al. [71] proposed estimating the volumetric heatmaps in an encoder–decoder manner. They first produced compressed volumetric heatmaps, which were used as ground truth, and then decompressed at test time to re-obtain the original representation. Zhen et al. [33] proposed estimating 2.5D representations of body parts first and then reconstructed the 3D human pose in a single-shot bottom-up framework. Wang et al. [74] also proposed distribution-aware single-stage models to represent 3D poses with a 2.5D human center, together with 3D center-relative joint offsets in a one pass solution.

TDBU_Net framework [36] combined top-down and bottom-up pipelines to accomplish the multi-person camera-centric 3D human pose estimation.

In this article, we were inspired by all of these proposals in building a top-down framework that could be used in real-world applications. We used monocular video as input, as in [35,36]. Thus, to deal with long-term models, we chose dilated temporal convolutional networks which only required the next images to produce real-time outputs. To respect this constraint, we also needed a system that integrated as few models as possible, unlike [35,36], while maintaining the highest possible accuracy.

## 3. Framework Overview

The first part of this section presents the basic architectures used in our framework, consisting of four phases: the human detector using Yolo-v3 architecture [75], the 2D human pose estimator employing HrNet network [23], the 3D root-relative pose estimator using the GAST-Net model [76], and the depth root estimator with the RootNet model [31]. The second part describes the overall pipeline of the framework. The last part details the series of enhancements of our framework on the 3D absolute pose estimator and their impacts on the final result.

### 3.1. Basic Models Architectures

**Human detector (Yolo-v3)**: This architecture [75] predicts bounding boxes using dimension clusters as anchor boxes. The network predicts four coordinates for each bounding box (bbox): the 2D image coordinates of the top-left pixel of the bbox, the width and height of the bbox, and the confidence score. Darknet-53 was used for feature extraction.

**2D pose estimator (HrNet)**: The high-resolution network [23] starts from a high-resolution subnetwork and gradually adds high-to-low resolution subnetworks one by one, by decreasing the resolution to half and increasing the width to double in separate branches that connect in parallel. In that way, high-resolution representation is maintained throughout the process. The input image size is 256 × 192 or 384 × 288, which produces 17 heatmaps (heatmap per each keypoint) of size 64 × 48 or 96 × 72 respectively. The authors proposed a small network (HRNet-W32) with 32 channels and a large one (HRNet-W48) with 48 channels.

**3D root-relative pose estimator (GAST-Net)**: The majority of models that recently analyzed and interpreted input video were based on temporal convolutional networks (TCNs), which were initially introduced to action segmentation by Lea et al. [55]. The GAST-Net (graph attention spatiotemporal network) [76] is inspired by VideoPose3D [39]. The network predicts 3D poses from 2D keypoints. It is designed from dilated temporal convolutional networks (TCNs) to tackle long-term patterns and exhibit extended memory, and from a graph attention block that consists of two spatial attention networks. The local spatial attention network models the hierarchical and symmetrical structures of the human skeleton. The global spatial attention network adaptively extracts global semantic information to better encode the spatial characteristics of the human body.

**Depth estimator (RootNet):** Moon et al. [31] proposed a top-down system to estimate 3D multi-person poses from a single RGB image, consisting of human detection by the DetectNet model, absolute 3D human root localization by the RootNet model, and root-relative 3D single-person pose estimation by the PoseNet model. Both models adopt ResNet-50 pre-trained on the ImageNet dataset as a backbone to extract the global data. We are particularly interested in the RootNet model, which generates two outputs: the 2D image coordinates of the root’s keypoint (x,y) estimated using soft-argmax on the root-heatmap (the central point of the individual), and the root depth absolute determined using a scalar value *k*, computed using focal lengths divided by the per-pixel distance factors and the human area ratio between the real-world and the image.

### 3.2. Taxonomy of the Framework

Given a sequence of bounding boxes from monocular RGB videos of a person or a group of people in real-time, the goal was to produce a sequence of 3D camera-centric coordinates of everyone in the scene. First, for each person, we assigned a unique ID *i* to be tracked through the successive frames. Then, we applied a high-resolution network (HRNet) [23] on each frame to produce 17 heatmaps. Each heatmap predicts 2D human joint locations in MS-COCO format $P_{2D}$ for each detected individual.

The 2D-poses $P_{2D}^{i}$ in 27 frames were collected and given thereafter to a 3D single-pose estimator, GAST-Net, for direct 2D-to-3D mapping and recovering of the 3D root-relative pose $P_{3Drel}^{i}$, where all produced joints were represented by their distances from the pelvis keypoints. GAST-Net was applied (as much as the number of people in the frame).

GAST-Net was chosen since it provides the best compromise between the number of frames required to process and the estimation precision. In fact, the methods with the best accuracies on monocular videos from Human3.6M (the largest database of 3D human pose estimation) are: temporal convolution [39] trained in semi-supervision learning, the Attention 3D Human Pose [77], which identifies significant frames and tensor outputs from each layer using the attention mechanism, the RIE paper [43], which improves the accuracy by relative information encoding that yields positional and temporal-enhanced representations, and Anatomy3D [78], which estimates the 3D skeleton by predicting bone orientation and length. These methods reached the MPJPEs (defined in Section 4) of 44.1, 43.3, 45.1, and 46.8 mm, respectively, but required 243 frames as input. This is very costly in terms of memory and processing time; moreover, this increases the delay between the image display and the result, which is not favorable for real-time processing. Furthermore, tracking several individuals on large time scales is more complicated and error-prone. On the other hand, approaches that employ few frames have higher errors. For example, VIBE [79] only used 16 frames but attained an MPJPE error of 65.6 mm, as well as TP-Net [80] which required 20 frames but had an average error of 52.1. Trajectory space factorization [41] scored an error of 46.6 mm from 50 frames; GAST-Net achieved an MPJPE of 46.2 mm using 27 frames. Thus, it presents a good compromise for use in real-world contexts.

For absolute depth estimation of the pelvis keypoint, we employed the RootNet network proposed in [31], due to its adaptability to any 3D root-relative estimator.

**Figure 3:**
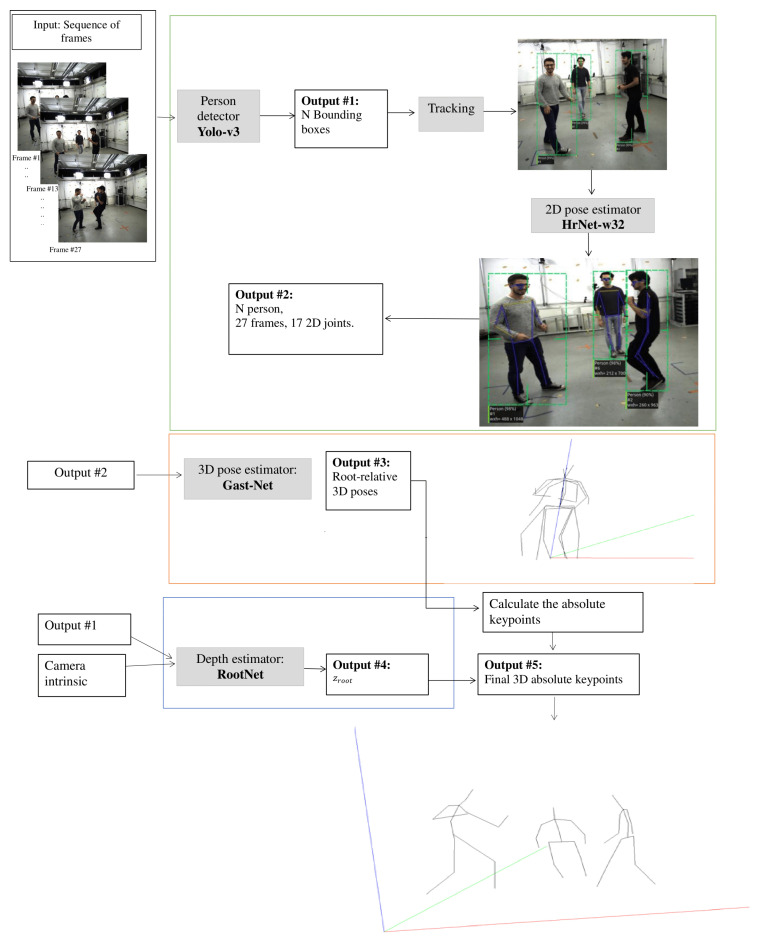
The pipeline of the Root-GAST-Net framework.

The proposed overall pipeline for estimating the absolute camera-centered coordinates of multi-person keypoints from a monocular camera is depicted in Figure 3. The pipeline comprises three boxes. Person detection and 2D keypoint estimation are included in the first box (green). The second box (orange) contains the 2D to 3D lift, and the last box (blue) contains the depth estimation.

### 3.3. 3D Absolute Pose Estimator

**Figure 4:**
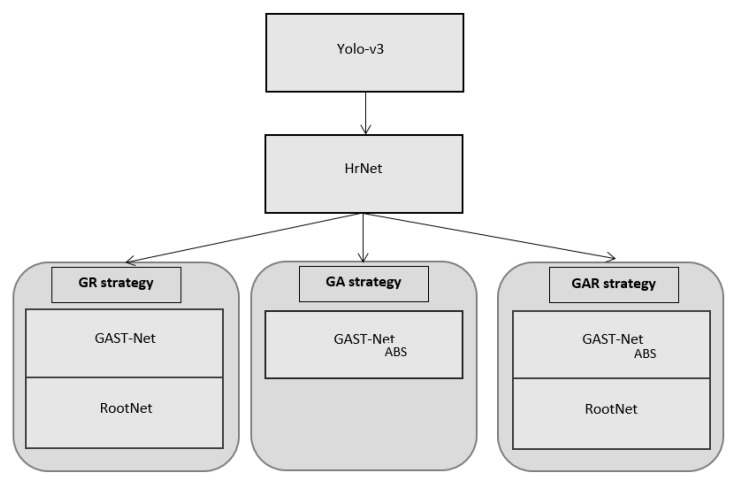
The structural diagram of the various types of networks used in the framework.

The purpose of this work was to develop a 3D multi-person camera-centric pose estimation system under industrial and real-world settings. Therefore, we started with a hybridization of well-chosen models, GAST-Net for predicting 3D root-relative keypoints and a RootNet network proposed in [31] for predicting absolute root depth (i.e., the depth of pelvis keypoint), obtained by multiplying *k* (defined above) by the scalar value of the network output. Then, the XY camera coordinates of the root were determined using the camera-intrinsic parameters, the image coordinates of the root, and the predicted absolute root depth. Finally, the absolute coordinates of the rest of the joints were estimated from these two predictions. We call this hybridization the GR method. On the MuPoTS-3D dataset, the system adopting the GR method outperformed previous methods by more than 12.1 percentage points on AP${}_{25}^{root}$, contributing to more than 6.7 percentage points on 3D-PCK${}_{abs}$. However, we observed that the root-relative keypoints were less good by 25.8 percentage points on PCK, which sparked the idea to upgrade the GAST-Net. While the original GAST-Net was trained on single-person databases [29], we chose to retrain our model on both a single-person video database (MPII-3DHP [81]) and a multi-person video database (MuCo-Temp [56]) with the required processing, following [56], to produce direct absolute keypoint coordinates. The TCN-based approaches evaluated on MuPoTS-3D were trained on the MPII-3DHP database, containing videos of a single person recorded in a green-screen studio and/or on the MuCo-3DHP database, composed of MPII-3DHP frames, containing multiple positions copied into a single frame. For this, in order to train the temporal networks, such as GAST-NET${}_{ABS}$,^56^] proposed MuCo-Temp, a temporal extension of MuCo-3DHP that was generated, such as MuCo-3DHP, but it is composed of videos instead of frames. As a result, the relative keypoint precision enhanced from 63.8% with the basic GAST-Net to 82.5% on PCK with our modified GAST-Net, which contributes to 1.6 percentage points in absolute points on 3D-PCK${}_{abs}$ when compared to the first methodology of hybridization. Note that in the following we name the upgraded GAST-Net by GAST-NET${}_{ABS}$, and this methodology by the GA method. We noticed that although AP${}_{25}^{root}$ of GAST-NET${}_{ABS}$ (measuring the root depth estimation) has improved compared to the state-of-the-art, it is still not as good as the first hybridization methodology. This pushed us to compute the root-relative keypoints from the absolute keypoints obtained by GAST-NET${}_{ABS}$ and employ the RootNet for root depth estimation, generating final absolute joints. We call this methodology the GAR method. In this way, we increased the accuracy (compared to the literature approaches) by more than 8.8 percentage points on 3D-PCK${}_{abs}$. Figure 4 presents the structural diagram of the various types of networks used in the framework.

All these experimental results will be presented, detailed, and analyzed in the next section (Section 4).

## 4. Experimentation and Results Discussion

This section deals with the experimental details and results of the proposed system. Results are discussed and evaluated using MPJPE, MRPE, 3D-PCK, AP${}_{25}^{root}$, 3D-PCK${}_{abs}$ metrics and response times. The proposed Root-GAST-Net system and its three variants (GR, GA, GAR), 3D pose absolute methodologies, were compared to the existing methods grouped in papers_With_Code link of 3D multi-person pose estimations (absolutes) on the MuPoTS-3D page (https://paperswithcode.com/sota/3d-multi-person-pose-estimation-absolute-on, accessed on 1 April 2022). The compared methods are 3D MPPE PoseNet [31], HDNet [32], SMAP [33], HMOR [69], GnTCN [35], and TDBU_Net [36]. The goal of evaluating the three methodologies was to measure the impact of each adjustment.

### 4.1. Datasets and Evaluation Metrics

**Human3.6M** is the most popular and biggest dataset/benchmark for 3D human pose estimation [29]. It contains 3.6 million single-person indoor video frames and the corresponding poses of 11 professional actors (6 males, 5 females) captured by the MoCap system from 4 camera viewpoints. Camera extrinsic (rotation and translation with respect to world coordinates) and intrinsic parameters (focal length and principal point) are also available. This could be used to evaluate the single-person-centric pose estimate [39,41,43,57,77,78,79,80] as well as the camera-centered coordinate prediction [31,32,33,35,36,69]. Only subjects 9 and 11 were used for testing, as in prior studies.

For evaluation, we computed the mean per joint position error metric (MPJPE), which is the mean Euclidean error averaged over all joints and all poses, calculated after aligning the human root of the estimated and ground truth 3D poses, calculated on relative poses, as shown in the formula below:

(1) $$MPJPE=\frac{1}{T}\frac{1}{N}\sum_{t=1}^{T}\sum_{i=1}^{N}{∥{J_{i}^{\left(t\right)}-J_{i}}^{*(t)}∥}_{2},$$

where *T* denotes the total number of test samples and *N* denotes the number of joints. *J* and $J^{*}$ denote the predicted joint and the ground truth joint, respectively.

Another evaluation metric used in this database, proposed in [31], is the mean root position error (MRPE), which is the average error of the absolute root joint (the hip) localization, as follows:

(2) $$MRPE=\frac{1}{T}\sum_{t=1}^{T}{∥(R^{\left(t\right)}-R^{*(t)})∥}_{2},$$

where *R* and $R^{*}$ denote the predicted root joint and the ground truth root joint respectively.

**MuCo-3DHP and MuPoTS-3D** MuCo-3DHP and MuPoTS-3D are two datasets proposed by Mehta et al. [34] for 3D multi-person pose estimation evaluation. MuCo-3DHP is the training dataset that merges randomly sampled 3D poses from a single-person 3D human pose dataset MPI-INF-3DHP [81] to form realistic multi-person scenes. MuPoTS-3D is a dataset used for testing 3D multi-person estimation. It contains 20 videos in both indoor and outdoor scenes. Ground truth is obtained with a multi-view markerless motion capture system.

In order to evaluate person-centric pose estimations, we used the percentage of a correct 3D keypoint (3D-PCK), which treats an estimated joint as correct if it is within a fixed threshold distance from the matched ground truth joint. In the literature, the threshold is set to 15 cm. We also used AUC${}_{rel}$, which is the area under the 3D-PCK curve computed from various thresholds.

We followed [31] to evaluate the absolute camera-centered coordinate estimations. We used average precision AP${}_{25}^{root}$ to measure the 3D human root location prediction error, which considers the prediction as correct when the Euclidean distance between the estimated and the ground truth coordinates is smaller than 25 cm. Moreover, we used 3D-PCK${}_{abs}$, which is PCK without the root alignment used to evaluate the absolute poses.

**MuCo-Temp** This dataset was proposed by [56]. It is generated in the same way as MuCo-3DHP. Both use images composited from the MPI-INF-3DHP dataset. The difference is that MuCo-Temp consists of videos instead of frames. So we can use it for temporal network training.

### 4.2. Implementation Details

We adopted Yolo-v3 architecture [75], which is based on the Darknet-53 model as a backbone and is pre-trained on the COCO dataset [82]. The input resolution is 608 × 608.

The cropped image of the bounding box was transformed to 384 × 288 to be used as input for the 2D pose estimator. The transformation applied was an affine transformation that preserves collinearity, parallelism, and the ratio of distances between the points, as in [23]. A unique ID was assigned to each person using the tracking method [83] based on the Hungarian optimization algorithm. Then, we used the small architecture of HrNet (HRNet-w32) pre-trained on the COCO dataset [82], implemented in PyTorch. The output was 17 heatmaps (resolution: 96 × 72). Cropping was resized to 256 × 256 to be processed by RootNet for depth root prediction $Z_{abs}^{root}$. A unique ID was affected for each person using the tracking method based on the Hungarian optimization algorithm. The 27 consecutive 2D coordinates were collected for each person, to be given to GAST-NET.

All networks, except GAST-NET, were optimized to TensorRT (https://developer.nvidia.com/tensorrt, accessed on 1 April 2022), a Nvidia library allowing to optimize computations on the GPU in order to reach lower computation times. This library also offers lower precision arithmetic but in our experiments, we kept models in the FP32 precision.

For GAST-NET${}_{ABS}$ training, we used the Adam optimizer with a learning rate of 1 × 10${}^{-3}$ and a batch size of 32. We trained the model for 80 epochs on MPII-3DHP [81] and MuCo-Temp [56] datasets. Computations were performed at the supercomputer facilities at Mésocentre Clermont Auvergne University for one week.

Finally, the detected bounding box was resized to 256 × 256 to be processed by RootNet for depth root prediction $Z_{abs}^{root}$.

### 4.3. Results

#### 4.3.1. Evaluation of Multi-Person Dataset MuPoTS

The results of our system with the three improvements are listed in Table 1, which can be compared to the literature results. We evaluated using the MuPoTS-3D dataset since it has been used to analyze 3D multi-person poses in both person-centric and camera-centric coordinates. Following [31,35], the performance of person-centric 3D pose estimation was evaluated using AUC${}_{rel}$ and PCK metrics, while camera-centric 3D pose estimation was evaluated using AP${}_{25}^{root}$ and PCK${}_{abs}$ metrics. The detailed PCK${}_{abs}$ results per sequence are shown in Table 2. We observed an improvement in the estimation accuracy in most of the sequences.

According to both tables, all our strategies outperformed previous 3D multi-person absolute pose estimation approaches by a significant margin, even if the relative poses were weaker.

**Table 1:** Person-centric and camera-centric evaluations on the MuPoTS-3D dataset. The best is in bold, the second best is underlined.

Method	Year	PCK	AUC${}_{\mathrm{rel}}$	3D-PCK${}_{\mathrm{abs}}$	AP${}_{25}^{\mathrm{root}}$
3D MPPE PoseNet [31]	2019	81.8	39.8	31.5	31.0
HDNet [32]	2020	83.7	-	35.2	39.4
SMAP [33]	2020	80.5	45.5	38.7	45.5
HMOR [69]	2020	82.0	43.5	43.8	-
GnTCN [35]	2021	87.5	48.9	45.7	45.2
TDBU_Net [36]	2021	**89.6**	**50.6**	48.0	46.3
DAS [74]	2022	82.7	-	39.2	-
Root-GAST with GR	-	63.8	30.6	54.7	58.4
Root-GAST with GA	-	82.5	45.3	56.1	56.8
Root-GAST with GAR	-	82.5	45.3	**56.8**	**58.9**

**Table 2:** Sequence-wise 3D-PCK${}_{abs}$ comparison with the state-of-the-art on the MuPoTS-3D dataset. (*) The accuracies of methods are measured on matched ground truths. The best is in bold, the second best is underlined.

**Method**	**S1**	**S2**	**S3**	**S4**	**S5**	**S6**	**S7**
3D MPPE PoseNet (*) [31]	59.5	45.3	51.4	46.2	53.0	27.4	23.7
HDNet [32]	21.4	22.7	58.3	27.5	37.3	12.2	49.2
SMAP (*) [33]	42.1	41.4	46.5	16.3	53.0	26.4	47.5
GnTCN (*) [35]	64.7	59.3	59.4	63.1	52.6	42.7	31.9
TDBU_Net [36]	69.2	57.1	49.3	68.9	55.1	36.1	49.4
Root-GAST with GAR (*)	**89.8**	**77.0**	**73.4**	**77.0**	**81.0**	**54.3**	**68.4**
**Method**	**S8**	**S9**	**S10**	**S11**	**S12**	**S13**	**S14**
3D MPPE PoseNet (*) [31]	26.4	39.1	23.6	8.3	14.9	38.2	29.5
HDNet [32]	40.8	53.1	43.9	43.2	**43.6**	39.7	28.3
SMAP (*) [33]	18.7	36.7	**73.5**	46.0	22.7	24.3	38.9
GnTCN (*) [35]	35.2	53.0	28.3	37.6	26.7	46.3	44.5
TDBU_Net [36]	33.0	43.5	52.8	**48.8**	36.5	51.2	37.1
Root-GAST with GAR (*)	**60.5**	**71.3**	65.4	33.5	26.1	**67.3**	**46.9**
**Method**	**S15**	**S16**	**S17**	**S18**	**S19**	**S20**	**Avg**
3D MPPE PoseNet (*) [31]	36.8	23.6	14.4	20.0	18.8	25.4	31.8
HDNet [32]	49.5	23.8	18.0	26.9	25.0	38.8	35.2
SMAP (*) [33]	47.5	34.2	35.0	20.0	38.7	64.8	38.7
GnTCN (*) [35]	50.2	47.9	39.4	23.5	**61.0**	**56.1**	46.3
TDBU_Net [36]	47.3	**52.0**	20.3	**43.7**	57.5	50.4	48.0
Root-GAST with GAR (*)	**66.9**	35.7	**40.1**	38.5	26.0	35.3	**56.8**

The average precisions throughout the entire dataset were then examined using various threshold settings ranging from 25 to 10 cm. AP measured the accuracy of the root key point; we only evaluated the Root-GAST system’s performance using the GA approach since GR and GAR methodologies employed RootNet to predict the root joint. They produced the same result as the original paper. Table 3 displays the results. When compared to the state-of-the-art methodology, our method significantly achieves greater AP across all levels of thresholds. We deduce that our method estimates many more correct root keypoints even with a low distance threshold.

To compare with most of the existing methods that evaluate person-centric 3D pose estimations on MuPoTS-3D using MPJPE, we report our results using the same metric in Table 4. Our result was 101.9 mm, the result of [34] was 132 mm, the result of [84] was 120 mm, the result of [56] when adding the pose refinement model was 103 mm. Our method also outperforms the existing methods on this metric.

#### 4.3.2. Evaluation on Single-Person Dataset Human3.6M

In order to validate the system, we chose Human3.6M, which contains only single-person videos. Since we compared the results through the mean root position error (MRPE) metric, which measured the accuracy error of the root key point, we only evaluated the Root-GAST system’s performance using the GA approach. GR and GAR methodologies employed RootNet to predict the root joint; they produced the same result as the original paper.

The root localization results of our GAST-Net${}_{ABS}$ and the RootNet model are shown in Table 5. Even though the evaluation was performed on the Human3.6M dataset, we employed the GA model that was retrained on MPII and the MuCo-Temp dataset, and we compared it to the RootNet model that was trained on the MuCo dataset to make a fair comparison. Our measurement error amounted to 158 mm, while that of [31] was 289.28 mm. However, we could expect greater improvement if we train our model in the Human3.6M dataset.

#### 4.3.3. Response Time

The response time is the processing time taken by the algorithm to process its input; it depends on the material configurations. The Root-GAST-Net pipeline was implemented in C++ and executed on a machine equipped with Intel Core i5-9500, with a dedicated memory of 32GB, and the Nvidia GeForce GTX 1080, with a dedicated memory of 8GB.

A comparative analysis of the response times of each network is shown in Table 6. The processing time was measured on batches of monocular images from the Human3.6M dataset, each containing one person. Note that the processing time of the tracking step is negligible.

Finally, the frame rate of the whole pipeline with each strategy is given in Table 7. The proposed Root-GAST-Net system can run at about 15 frames per second, which is suitable for real-time scenarios. Therefore, improving the metrics does not impact the real-time aspect of the pipeline.

#### 4.3.4. Qualitative Results

**Figure 5:**
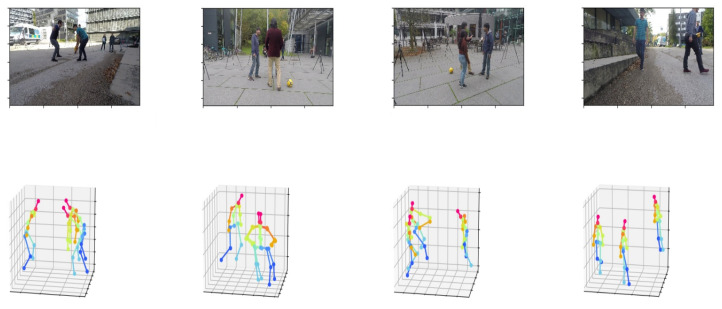
Erroneous 3D multi-person pose estimation. The first two images represent two similar poses of different people because one is completely occluded. In the right two images, one pose is incorrect because the body parts are partially outside of the boxes.

As the system follows a top-down approach, the final result depends on all previous outputs. If the detection is not correctly done, the 2D keypoints and depths will be wrongly estimated, which will impact the absolute pose. If there are numerous people inside the box or body parts that are partially outside the box’s bounds, the full-body joint calculation is likely to be incorrect, as shown in Figure 5. The confusion stems from erroneous 2D point estimations, which have negative impacts on the 3D-lifted process.

## 5. Conclusions

In this work, we propose a top-down framework for 3D multi-person absolute pose estimation, reconstructed from 2D poses from a monocular camera. Our framework Root-GAST-Net can combine different models in three strategies. The GR strategy and GAR strategy, which integrate human detection, 2D pose estimation, 3D human root-relative single-person pose estimation, and root depth estimation. Moreover, the GA strategy integrates human detection, 2D pose estimation, and 3D absolute pose estimation.

Experimental results on multiple datasets showed that our framework significantly outperforms the recent approaches in 3D absolute multi-pose estimation. In addition, the system can be used in real-time, as the execution time of each frame containing one person takes around 60 milliseconds using the Nvidia GeForce GTX 1080. This can be reduced using high-performance materials and FP16 precision.

In future works, we plan to retrain the model on the Human3.6M dataset to improve the evaluation accuracy of this database. We also plan to develop a fall detection application based on the absolute and relative 3D postures predicted by the Root-GAST-Net system.

## Figures and Tables

**Table 3:** Average precision of the root keypoint evaluation by different distances on the MuPoTS-3D dataset.

Method	AP${}_{25}^{\mathrm{root}}$	AP${}_{20}^{\mathrm{root}}$	AP${}_{15}^{\mathrm{root}}$	AP${}_{10}^{\mathrm{root}}$
3D MPPE PoseNet [31]	31.0	21.5	10.2	2.3
HDNet [32]	39.4	28.0	14.6	4.1
Root-GAST with GA	56.8	47.1	36.8	22.4

**Table 4:** MPJPE of the relative poses on the MuPoTS-3D dataset. The best is in bold, the second best is underlined.

Method	Year	MPJPE (mm)
Temporal smoothing [56]	2020	107
Temporal smoothing + Pose refinement [56]	2020	103
Depth Prediction Network [84]	2019	120
LCR-Net [67]	2017	146
Mehta et al. [34]	2018	132
GAST-Net${}_{ABS}$	-	**101.9**

**Table 5:** MRPE results comparison with RootNet [31] on the Human3.6M dataset. MRPE${}_{x}$, MRPE${}_{y}$, and MRPE${}_{z}$ are the average MRPE errors in the *x*, *y*, and *z* axes, respectively.

Method	MRPE (mm)	MRPE${}_{x}$ (mm)	MRPE${}_{y}$ (mm)	MRPE${}_{z}$ (mm)
3D MPPE PoseNet [31]	289.28	35.95	58.65	268.49
Root-GAST with GA	178	33	41.9	158

**Table 6:** Response time per model.

Model	Min Response Time (ms)	Max Response Time (ms)	Average Response Time (ms)
Yolo-v3	24	30	28
HrNet	9	12	10
GAST-Net	27	33	29
GAST-Net${}_{ABS}$	23	29	26
RootNet	4	8	5

**Table 7:** Frame rate per strategy.

Strategy	Average Frame Rate (fps)
Root-GAST with GR	13
Root-GAST with GA	16
Root-GAST with GAR	15

## Data Availability

We used the MuCo-Temp dataset, generated with the GitHub project at pose_refinement (https://github.com/vegesm/pose_refinement (accessed on 12 February 2022)) for training. For evaluation, we used the Human 3.6M dataset, parsed and available at 3DMPPE_ROOTNET_RELEASE github project https://github.com/mks0601/3DMPPE_ROOTNET_RELEASE (accessed on 12 February 2022), and the MuPoTS-3D dataset is publicly available at website https://vcai.mpi-inf.mpg.de/projects/SingleShotMultiPerson/ (accessed on 12 February 2022).

## Abbreviations

The following abbreviations are used in this manuscript:

HPE	human pose estimation
LSTM	long short-term memory
GNN	graph neural network
GCN	graph convolution network
TCN	temporal convolutional network
RNN	recurrent neural network
MPJPE	mean per joint position error
MRPE	mean of the root position error
AUC	area under the curve
3D-PCK	percentage of correct key-points in 3D space
AP${}^{root}$	average precision of the root keypoint
GPU	graphics processing unit
GR	first 3D absolute pose methodology: GAST-Net + RootNet
GA	second 3D absolute pose methodology: GAST-Net${}_{ABS}$ trained on MuCo-Temp
GAR	third 3D absolute pose methodology: GAST-Net${}_{ABS}$ trained on MuCo-Temp + RootNet
Root-GAST	the whole pipeline: human detector + 2D pose estimator + 3D absolute pose estimator
